# Rats Synchronize Locomotion with Ultrasonic Vocalizations at the Subsecond Time Scale

**DOI:** 10.3389/fnbeh.2016.00184

**Published:** 2016-09-29

**Authors:** Diego A. Laplagne, Martín Elías Costa

**Affiliations:** ^1^Laboratory of Behavioral Neurophysiology, Brain Institute, Federal University of Rio Grande do NorteNatal, Brazil; ^2^Department of Physics, University of Buenos AiresCiudad de Buenos Aires, Argentina

**Keywords:** rat, ultrasonic vocalizations, locomotion, behavior, animal communication, audience effect, contact calls

## Abstract

Acoustic signals have the potential for transmitting information fast across distances. Rats emit ultrasonic vocalizations of two distinct classes: “22-kHz” or “alarm” calls and “50-kHz” calls. The latter comprises brief sounds in the 30–80-kHz range, whose ethological role is not fully understood. We recorded ultrasonic vocalizations from pairs of rats freely behaving in neighboring but separated arenas. 50-kHz vocalizations in this condition were tightly linked to the locomotion of the emitter at the subsecond time scale, their rate sharply increasing and decreasing prior to the onset and offset of movement respectively. This locomotion-linked vocalization behavior showed a clear “audience effect,” as rats recorded alone displayed lower vocal production than rats in social settings for equivalent speeds of locomotion. Furthermore, calls from different categories across the 50 and 22-kHz families displayed markedly different correlations with locomotor activity. Our results show that rat vocalizations in the high ultrasonic range are social signals carrying spatial information about the emitter and highlight the possibility that they may play a role in the social coordination of spatial behaviors.

## Introduction

Most mammals share with humans the ability of emitting vocalizations, stereotyped sounds produced by pushing air through constricted vocal folds in the larynx. These are controlled by sets of muscles and brain structures largely shared across species (Hoh, 2010; Newman, 2010). The majority of mammalian vocalizations, like human voice, are produced when air flowing out through tensed vocal folds causes them to vibrate resulting in sound pressure waves of rich harmonic content. Rats emit audible calls in this way in response to aggression, closely approaching predators or painful stimuli (Litvin et al., 2007). However, most of their vocal production happens outside of our hearing range through a different mechanism. It is currently believed that these ultrasonic vocalizations (USVs) are produced when air flowing through a small orifice formed by tight vocal folds produces nearly pure tones via an aerodynamic whistle mechanism (Roberts, 1975b; Riede, 2011).

Rat USVs fall in two families of calls with distinct ethological and neurophysiological correlates (reviewed in Brudzynski, 2009). Aversive settings such as the anticipation of pain or danger can result in prolonged emission of ultrasound in the 20–25-kHz range with little or no frequency modulation, named “22-kHz” USVs or “alarm calls.” These calls can be accompanied by extreme immobility (freezing), a typical fear response, and can in turn induce freezing or avoidance in listening rats (Kim et al., 2010; Parsana et al., 2012). Twenty-two kilohertz calls thus seem to effectively communicate fearful or anxious states to conspecifics.

The other family of rat ultrasonic calls, named “50-kHz” USVs, includes a variety of brief sounds (typically under 100 ms) with frequencies within 30–80-kHz. Their frequency can be relatively constant (“flat”), follow stereotyped modulation at ~100 Hz (“trill”) or present sudden jumps. Furthermore, these elements can be combined within single calls resulting in a potentially large “dictionary” of signals (Wright et al., 2010). Both male and female rats often emit high rates of 50-kHz calls in social settings such as mating or play (Sales, 1972; Thomas and Barfield, 1985; Knutson et al., 1998). Isolated rats will exhibit comparable rates if acutely expecting reward or social contact (Burgdorf et al., 2000; Brudzynski and Pniak, 2002) or upon direct experimental activation of the mesolimbic dopaminergic pathway (Burgdorf et al., 2007). At faster time scales, we have found that rats emit high rates of these USVs only during bouts of fast sniffing, part of their active exploratory behavior (Sirotin et al., 2014).

Despite the well-established correlations between 50-kHz call emission and behavioral or emotional states (Knutson et al., 2002; Brudzynski, 2015), the specific contributions of this USV family to rat social behavior are not fully understood. Rats have been shown to actively seek 50-kHz USVs, as playback of these calls can induce initial approach behavior and rats can self-administer them if given the possibility (Wöhr and Schwarting, 2007; Burgdorf et al., 2008; Willadsen et al., 2014). These results suggest that 50-kHz USVs could function as “contact calls” (Seffer et al., 2014). Devocalizing experiments, however, have not revealed a clear role of these USVs in social behaviors. Devocalizing either the male or the female in a mating pair did not alter the sexual behavior of males (Thomas et al., 1981; White and Barfield, 1987; Ågmo and Snoeren, 2015). In turn, these procedures affected only specific aspects of female solicitation in some works (Thomas et al., 1981; White and Barfield, 1987) but not in others (Ågmo and Snoeren, 2015). Strikingly, females did not approach vocalizing males more than they did devocalized ones, nor did they choose them more as mating partners (Snoeren and Ågmo, 2014a,b; Ågmo and Snoeren, 2015). The role of USVs in male dominance is also unclear, as devocalizing either intruder or resident males had no effect on aggressive or defensive behavior (Takahashi et al., 1983; Thomas et al., 1983).

Across vertebrates, contributions of contact calls to social behavior appear when individuals are within audible range but separated (Boinski, 1993; Rendall et al., 2000; Marler, 2004; Radford, 2004; Kondo and Watanabe, 2009). To gain insights into the ethological roles of 50-kHz USVs, we mimicked this condition in a laboratory setting by performing simultaneous audio and video recordings from pairs of rats (male/male and male/female) in the same acoustical environment but without physical or visual contact between them. We developed methodology to identify the emitter of the recorded calls and correlate their emission with spatial behavior with high temporal precision. This work details the unexpectedly precise synchrony between emission of 50-kHz USVs and locomotion displayed by all the rats. We further discuss possible implications of these findings on the behavioral roles of rat ultrasonic vocalizations.

## Materials and methods

### Animal subjects and recording sessions

**Figure 1:**
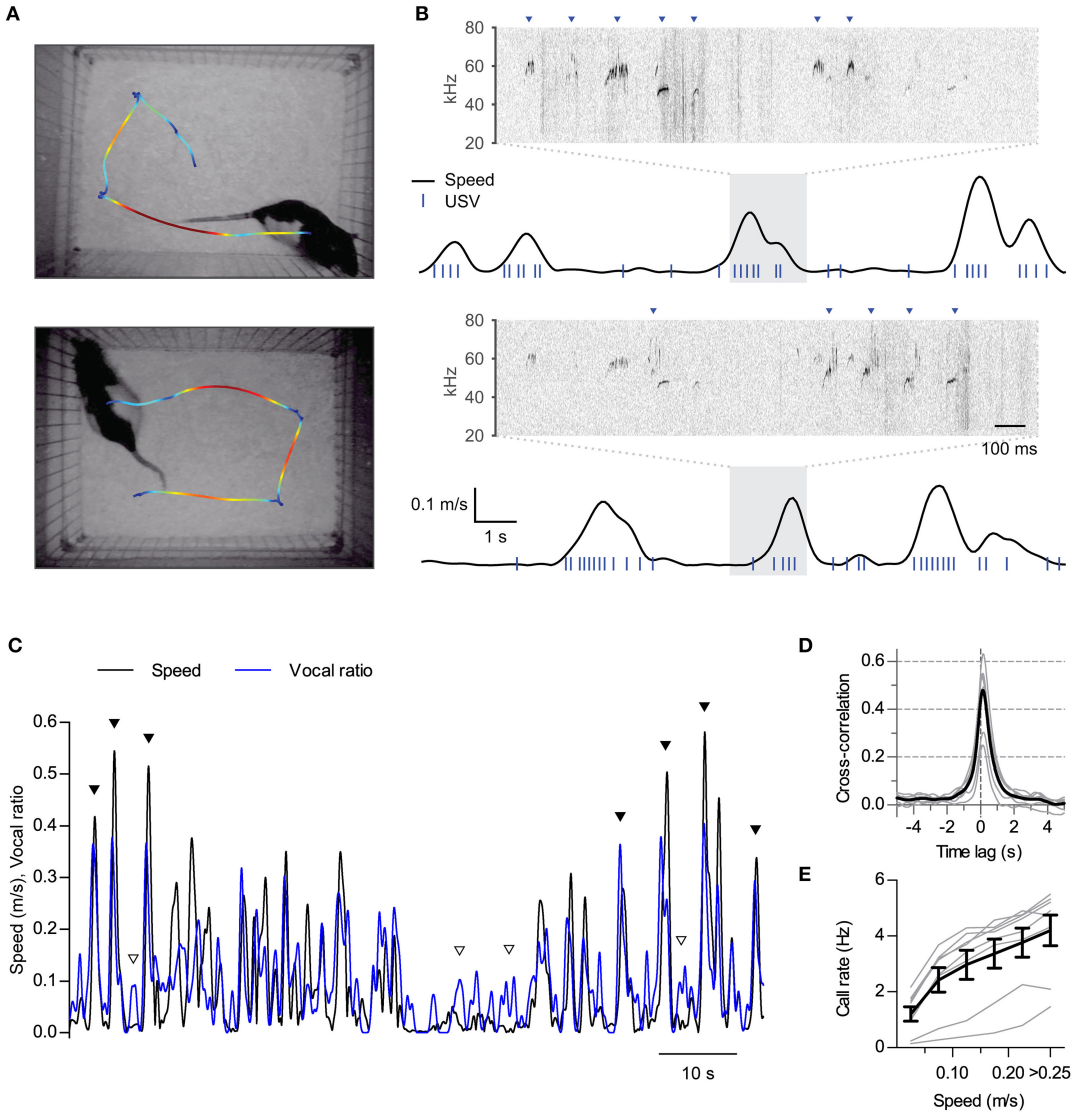
**Synchronization of ultrasonic vocalizations to locomotion**. **(A)** Locomotion of two rats during 16 s of interaction in the split social arena. The trajectory of each rat was overlaid on the final frame of the video sequence, with colors representing instantaneous speed (blue to red: 0–0.25 m/s). **(B)** Analysis of locomotion and vocal production for each rat during the same time period represented in **(A)** Black traces show instantaneous speed and blue ticks times when ultrasonic vocalizations were detected for each rat. Note vocalizations coincide with movements of the rats. Sonograms from the shaded times at each recording microphone are expanded on top of each plot. Arrowheads point to detected vocalizations assigned to each rat. **(C)** Instantaneous speed (black, m/s) and vocal production (blue, vocal ratio) during 90 s of recording from one rat during a social session. Filled and open arrowheads highlight examples of vocal production synchronous or not with locomotion episodes. **(D)** Cross-correlation of instantaneous speed and vocal ratio (gray: mean for each rat; black: grand mean across rats). Peak = 0.47 [0.37, 0.57], width at half maximum = 0.88 s [0.80, 0.96], lag of peak = 136 ms [112, 159]. Mean [95% CI]. **(E)** Mean call rate vs. instantaneous speed (gray: line for each rat; black: mean ± s.e.m. across rats).

**Figure 2:**
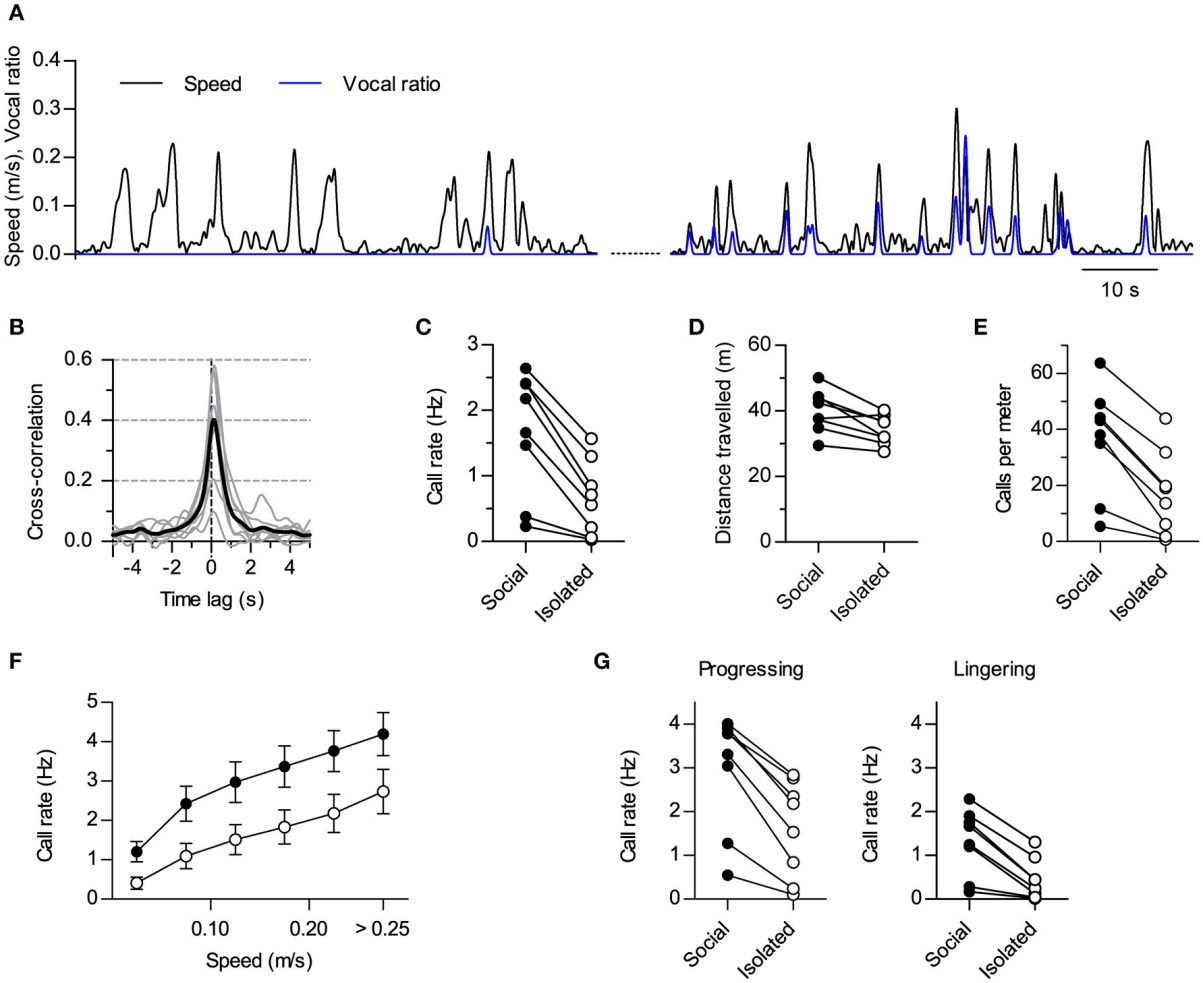
**Modulation of vocal production by social context. (A)** Instantaneous speed (black, m/s) and vocal production (blue, vocal ratio) of one rat during a session in isolation (compare with Figure 1C). Two periods from the same session are shown (70 s each; early on the left, late on the right). **(B)** Cross-correlation of instantaneous speed and vocal ratio (compare with Figure 1D; gray: mean for each rat; black: grand mean across rats). Peak = 0.43 [0.27, 0.59], width at half maximum = 1.01 s [0.76, 1.25], lag of peak = 130 ms [111, 150] (lag different from 0 with *p* < 0.0001, one sample *t*-test). *N* = 8 rats. **(C)** Mean call rate of each rat during social and isolated sessions. Social—Isolated mean difference = 1.0 Hz [0.59, 1.43]. **(D)** Mean distance traveled per 15 min session. Mean difference = 5.7 m [2.1, 9.2]. **(E)** Mean number of calls per meter traveled. Mean difference = 19 calls [12, 26]. **(F)** Call rate vs. instantaneous speed during social (filled) and isolated (open) sessions (grand mean ± s.e.m. across rats). **(G)** Mean call rate of each rat during progressing (left; mean difference = 1.4 Hz [0.9, 1.8]) and lingering (right; mean difference = 0.9 Hz [0.5, 1.2]) episodes for social and isolated sessions. Values in brackets: 95% CI.

**Figure 3:**
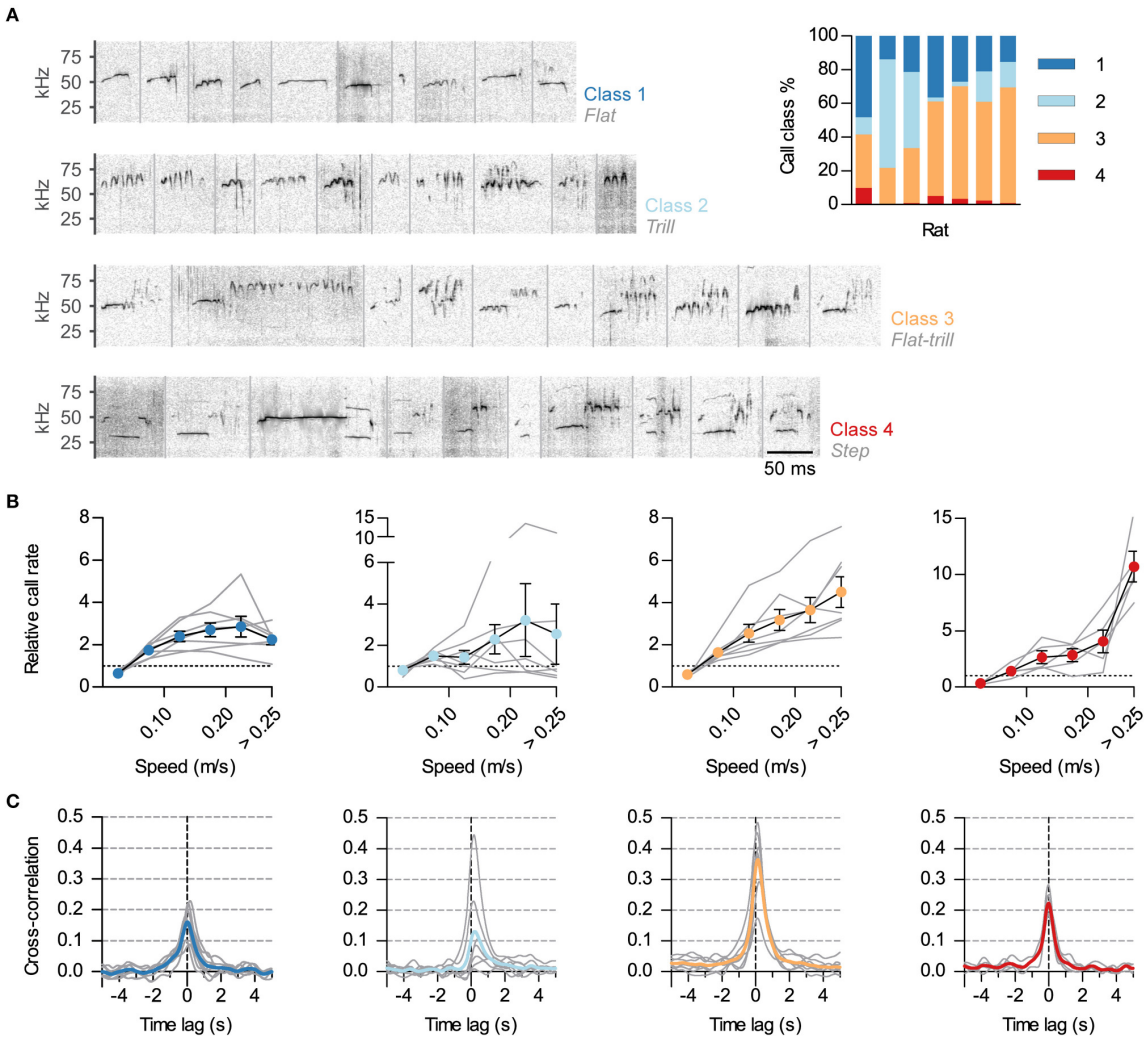
**Differential synchronization of call classes to locomotion. (A)** Calls were automatically classified into 4 classes based on their spectrotemporal profiles. Shown are example sonograms of 10 calls from each class, separated by gray vertical lines. Names of the previously described call categories most represented in each of the 4 classes are shown in gray under the class numbers. Inset: Call class usage for each of the 7 analyzed rats. **(B)** Relative call rate vs. instantaneous speed for each class (1–4, left to right; gray lines: rates for each rat, symbols: mean ± s.e.m. across rats). Rates were divided by the mean call rate for each class for each rat. Two rats were excluded from the analysis of class 4 as they emitted <5 of these USVs. Note different vertical scales. **(C)** Cross-correlation of instantaneous speed and vocal ratio for each class (gray: mean for each rat; color: grand mean across rats). Peaks (mean and 95% CI): class 1, 0.17 [0.12, 0.22]; class 2, 0.14 [0, 0.28]; class 3, 0.37 [0.27, 0.46]; class 4, 0.22 [0.17, 0.28].

**Figure 4:**
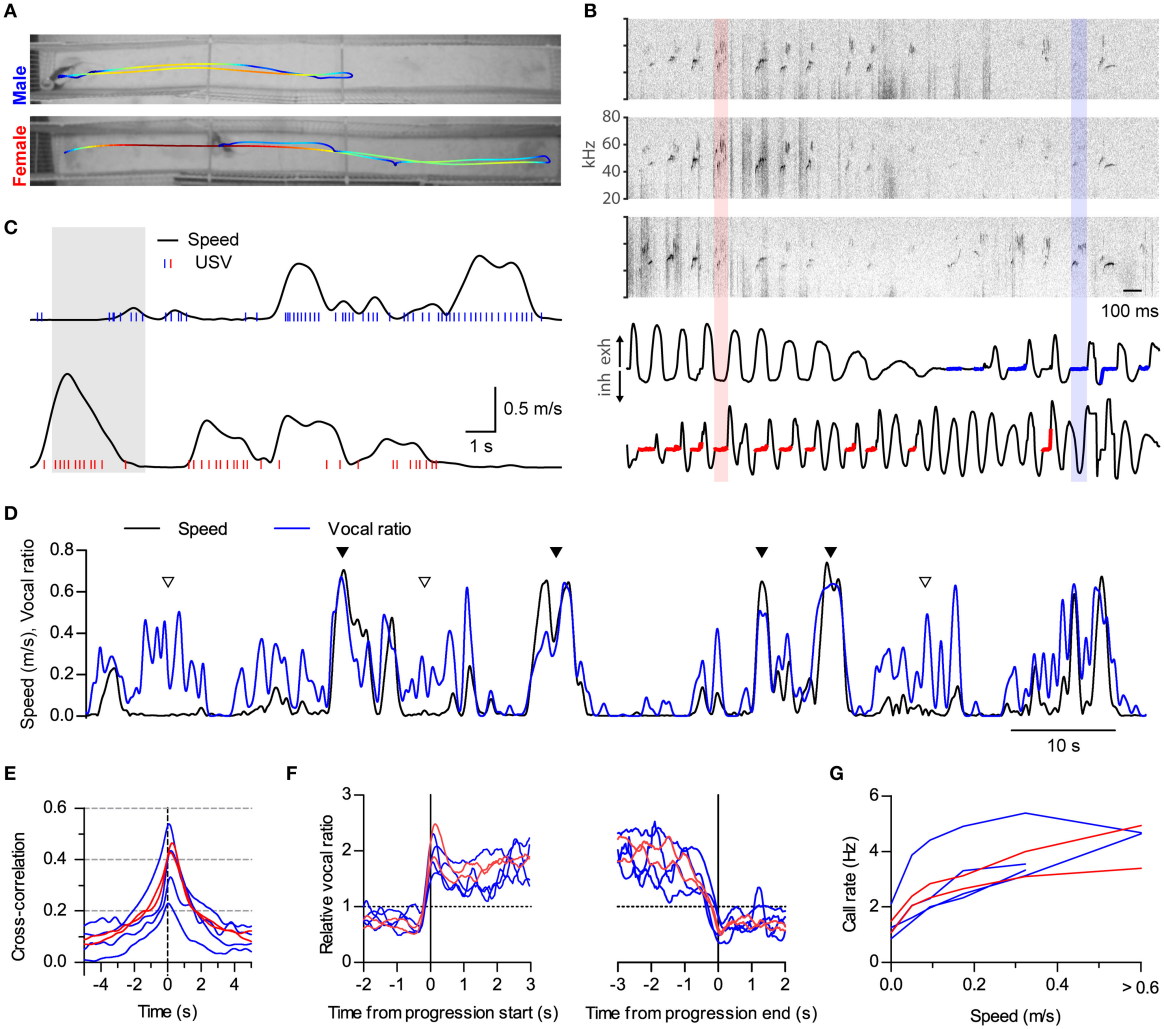
**Sustained high vocal production in prolonged fast progressions. (A)** Locomotion of two rats during 18 s of interaction in a 2.7 m long split social arena. The trajectory of each rat is overlaid on the final frame of the video sequence, with colors representing instantaneous speed (blue to red: 0–1.1 m/s). **(B)** Assignment of vocalizations based on respiration. Top: sonograms from the three microphones overhanging the arena during 3 s of the recording shown in (**A**; shaded times in **C**). Bottom: Intranasal pressure recorded at the same times from the male (top) and the female (bottom). Thick blue and red traces mark the times when USVs were assigned to the male or the female, respectively, based on their respiratory signal. One example from each is highlighted with shaded rectangles. **(C)** Analysis of locomotion and vocal production for each rat during the same time period represented in **(A)**. Black traces show instantaneous speed and blue/red ticks times when ultrasonic vocalizations were detected from the male/female. **(D)** Instantaneous speed (black, m/s) and vocal production (blue, vocal ratio) during 100 s of recording from a male rat during a social session. Filled and open arrowheads highlight examples of vocal production synchronous or not with locomotion episodes. **(E)** Cross-correlation of instantaneous speed and vocal ratio for each rat (blue: males; red: females). Peak = 0.40 [0.29, 0.52], width at half maximum = 2.43 s [2.08, 2.78], lag of peak = 165 ms [77, 252] (lag different from 0 with *p* < 0.01, one sample *t*-test). Mean [95% CI], *N* = 6 rats. **(F)** Mean relative vocal ratio for each rat aligned to the onset (left) and offset (right) of all its progressions (blue: males; red: females). Data before and after the progressions includes only lingering episodes within those 2 s windows. Call rates were higher during progressing than during lingering episodes (*p* < 0.001, two-tailed paired *t*-test, *N* = 6 rats). **(G)** Mean call rate vs. instantaneous speed for each rat (blue: males; red: females).

All procedures were approved by The Rockefeller University Institutional Animal Care and Use Committee (Protocol #09035). All animals (16 total) were Long Evans adult rats (Charles River), living in an animal facility located within 10 m of the recording room. Rats were held on an inverted light cycle and all recordings were carried out during the dark phase under infrared illumination. We habituated the animals through 5 min daily handling sessions for 5 days prior to experimental sessions. Results presented in this manuscript include recording sessions in two different social arenas: “small” (Figures 1–**3**) and “large” (**Figure 4**), all of which took place in a single-walled soundproof room.

#### Small arena

We used and thoroughly described this arena in Sirotin et al. (2014). The arena is built with vertical gratings and split in two halves, 0.46 × 0.33 × 0.74 m (W × L × H) each, 0.25 m apart on the wide side (Figure 1A). Results in the small arena were obtained from 8 adult male rats (2 groups of 4, housed in pairs; age at the time of recordings, group A: 12 weeks, group B: 9 weeks). Rats took part in “social” and “isolated” sessions, all of 15 min duration. In social sessions, one rat was placed on each side of the arena where they could hear and smell each other. In isolated sessions, only one rat was placed in the arena in a side chosen at random. All rats took part in 3 social sessions, one with each other rat in the group. In group A, each rat was recorded in isolation once. In group B, each rat was recorded in isolation 3 times. Social and isolated sessions were interleaved and balanced across days for each group, with each rat taking part of 1 social and 1 isolated session per day. For half the rats in each group their first session in the arena was a social one, while for the other half it was an isolated one. Results in Figures 1–**3** combine recordings from groups A and B.

#### Large arena

We recorded two females (ages 4.5–6.5 months) and four males (ages 2.5–4.5 months) in the large arena in a total of eight recording sessions involving five different rat pairings. We implanted these six rats (group C) with intranasal cannulae to record respiration. The estrous cycle of females was controlled through ovarectomy and hormonal treatment and all recordings were made during estrus. This arena was built with vertical gratings and split in two parallel linear tracks (see **Figure 4A**), 0.2 × 2.67 × 0.74 m (W × L × H) each, 0.15 m apart on the wide side. All sessions in this arena involved one male and one female, one on each side. In order to prime the animals for active courtship behavior during the recordings, these sessions were preceded by a brief interaction between male and female in a small cage that lasted up to the first mount with a maximum duration of 10 min. Rats in this arena carried a headstage for telemetric recording of respiration.

### Data acquisition

#### Video

We used webcams with infrared filters removed to record video. For rats in group A (small arena), we used Microsoft Lifecam VX-1000 yielding between 12 and 15 frames per second. For all others we used Logitech c920, which performs on-board video compression and stably yields 30 frames per second. We synchronized video with audio with <1 frame precision through an infrared LED blinking in the visual field of the cameras controlled by the DAQ board used for audio acquisition.

#### Ultrasound

We recorded ultrasound with condenser microphones with nearly flat (±5 dB) response from 10 to 150-kHz (CM16/CMPA-5V, Avisoft Bioacustics) digitized by a data acquisition board at 250-kHz sampling frequency (PCIe-6259 DAQ with BNC-2110 connector, National Instruments).

#### Respiration

For recordings in the large arena, we implanted rats with nasal cannulae bearing a ring magnet on the exposed end. During recordings, we magnetically attached a pressure sensor (24PCAFA6G, Honeywell) to the cannula that was integrated into a custom-made wireless headstage based on the DIGI XBee module (schematics available on request). The pressure signal was transmitted with a sampling rate of 100 Hz and digitized in synchrony with the ultrasound.

### Surgery and pharmacology

Rats who underwent surgery were anesthetized with a combination of ketamine, xylazine and atropine (i.m.; 100, 6, and 0.04 mg/kg, respectively). After surgery, buprenorphine (i.p.; 0.1 mg/kg) was administered as analgesic and enrofloxacin (i.p.; 20 mg/kg) as antibiotic. Animals recovered for at least 1 week before recordings.

We implanted rats destined for recordings in the large arena with intranasal cannulae to monitor respiration. As described in (Sirotin et al., 2014), the end of a thin 2-cm-long stainless steel cannula (gauge 22) was implanted through the nasal bone. The cannula was bent to an S-shape for it to end above the temporal bone and secured with bone screws and dental acrylic. A ring magnet (R422; OD 6.35 mm, ID 3.18 mm; K&J Magnetics) was attached to the exposed end of the cannula to match an equivalent one secured to the pressure sensor in the wireless headstage. This allowed us to easily and safely secure the headstages on the rats' heads by using magnetic force only.

For bilateral ovarectomy, we made incisions through the skin and muscle posterior to the rib cage, through which we pulled the ovaries out with forceps. After clamping the uterine horns with hemostats and absorbable suture we proceeded to cut off the ovaries, suture the muscle with absorbable thread and close the skin with suture clamps. For induction of estrus we injected females with estradiol benzoate (s.c.; 0.05 mg/kg) followed, 48 h later, by progesterone (s.c.; 2.5 mg/kg). Recordings were made 5–10 h after the administration of progesterone.

### Data pre-processing

We carried out all data pre-processing with custom-made routines in MATLAB (The Mathworks).

#### Locomotion

All of the analysis of locomotor activity was based on video tracking. For each time point in the recordings we obtained the position of the rat in the arena and, from the rate of change of this position, its instantaneous speed of locomotion. Examples of instantaneous speed time series can be seen in the black traces of Figures 1B,C, 2A, **4C,D**. Instantaneous speed of locomotion was typically between 0 m/s (when the rats were staying in place) and 1 m/s (during the fastest runs in the large arena). Throughout the paper, “speed” refers to this instantaneous speed of locomotion. In section Analysis of Vocalization vs. Locomotor Activity, we describe how we used this instantaneous speed to study the correlations between emission of USVs and locomotor activity.

##### Instantaneous position and speed

We obtained the position of the rat in each video frame through a custom-made implementation of object tracking based on adaptive background subtraction. Briefly, we selected a region of interest from the video and obtained an initial background image from the mean of 100 random frames. We then started the tracking by subtracting this background from the starting frame and computing the position of the rat as the center of mass of the difference values exceeding a fixed threshold. As the tracking progressed, we updated the background image in 1% with each new frame and computed the position of the rat in the same way. The background was not updated in a circle of diameter 30 cm around the position of the rat.

Before calculating instantaneous speed, we smoothed the position time series by independently convolving its two dimensions with a Gaussian window of 0.25 s (full width at half maximum). For each time point, we obtained the velocity vector as the derivative of each smoothed dimension and computed instantaneous speed as its norm.

##### Segmentation

For the analysis in Figures 2G, **4F**, we segmented locomotion into progressing and lingering episodes implementing the methods developed in Drai et al. (2000) and Hen et al. (2004). Briefly, we identified arrests by first applying two rounds of a running median filter on the two dimensions of the position time series: each value was replaced by the median of a 5-point window centered at it and the process was repeated using a 3-point window. We obtained an instantaneous speed from this filtered position and detected as “arrests” all stretches of at least 0.2 s with values under 0.05 m/s. We then marked each segment between two arrests as a “progression” if it lasted over 0.5 s and achieved a maximum speed of at least 0.1 m/s. Other segments were grouped together with interleaved arrests as “lingering” episodes.

#### Ultrasonic vocalizations

We used custom-made routines specific to the small and large arenas to automatically detect USVs and assign them to the emitting rat.

##### Small arena

Procedures for detecting and assigning USVs in this arena are described in (Sirotin et al., 2014). Briefly, we recorded ultrasound from two overhanging microphones, one on top of each side of the arena. We obtained sonograms for each microphone signal with 0.25 ms time step and detected times with low entropy of the frequency spectrum in the 18–100-kHz range. We then extracted as USVs segments of low entropy lasting at least 3 ms and bounded by silences of >20 ms. For each USV we compared the signals from both microphones at that time and assigned the emission of the USV to the rat on the side with lowest spectral entropy. When USVs were detected simultaneously at both microphones, we compared their spectrograms and, if different, we assigned one USV to each rat. We have estimated that 94% of USVs emitted in this arena are effectively detected and 99.8 ± 0.1 % of these are correctly assigned to the emitting rat (see Section Materials and Methods and Supplementary Figure 1 in Sirotin et al., 2014). We visually inspected the sonograms from all of the putative USVs and removed any noises detected as USVs by mistake. As an additional control, we manually detected USVs by visual inspection of the sonograms in 2 min segments from three social sessions and compared their numbers with those detected automatically. The number of USVs (combining those from both rats) detected in each segment were (manual vs. automatic): 483 vs. 484, 622 vs. 615, and 766 vs. 739.

##### Large arena

Given the size of this arena it was not feasible to use the previously described methodology to assign USVs to the emitting rat. We thus developed a new method based on the analysis of the respiratory cycle. As expanded below, we detected USVs with overhanging microphones and assigned each to the rat whose respiration at the time was compatible with vocalization.

During the emission of USVs, air pressure in the nasal cavity is maintained approximately constant at atmospheric values. Respiratory cycles with USV emission can thus be identified as those with a period of flat intranasal pressure immediately following inhalations (Supplementary Figures 1A,B; Sirotin et al., 2014). We recorded intranasal pressure from all rats in the large arena, as explained in Section Respiration. For each respiration recording we subtracted the atmospheric pressure baseline and transformed it to a z-score by normalizing it by its standard deviation.

We recorded ultrasound from 3 overhanging microphones distributed along the midline of the arena and detected times with low entropy on each. We extracted as USVs segments with low entropy in any microphone, bounded by silences of >20 ms in all of the microphones. In this arena, we did not resolve cases of simultaneous vocalization from the two rats, so only one rat could be considered to be vocalizing at any given time. We used a sequential set of criteria to assign each USV to the emitting rat. First, the algorithm measured the mean absolute intranasal pressure for each rat during the USV emission (Supplementary Figure 1A). If the difference between them was larger than 0.1 (*z*-score), it assigned the call to the rat with the lowest absolute pressure value (0 = atmospheric pressure). Otherwise, it attempted to disambiguate by looking at the flanking pressure traces (Supplementary Figure 1B). USVs should be preceded by negative pressure (inhalation) and followed by positive pressure (exhalation). We thus measured pressure in 50 ms windows immediately preceding and following the USV and subtracted the mean of the former from that of the latter for each rat. If for one of the rats this value was both >1.0 and larger than that of the other rat by at least 1.0, the method assigns the USV to it. If these criteria are not met, the USV is not assigned and removed from all further analysis.

We evaluated the accuracy of this method by recording from a rat alone in the large arena (22 min, ~2200 detected USVs, Supplementary Figures 1C–F). Errors in the assignment of a USV would arise in cases where, by chance, the respiration from a non-vocalizing rat at the time of the vocalization fits the described criteria better than that of the emitting rat. We simulated the respiration of another rat by circularly shifting the pressure signal of the recorded rat by a random amount of time and ran our assignment algorithm for the detected USVs on the real vs. the simulated respiration (ideally, 100% of the USVs should be assigned to the real rat). 92.8% of the calls were assigned by the algorithm, of which 98.2% were correctly assigned to the real rat. We expect the percentage of unassigned calls to increase with rats vocalizing at higher rates, as this would result in more simultaneous calls. We do not have a reliable estimate of what percentage of calls do not get detected in this large arena. However, most calls could be detected on more than one microphone, suggesting the setup was effectively picking up calls across the length of the arena.

### Analysis of vocalization vs. locomotor activity

We quantified the relationship between vocalizations and locomotor activity in three ways: (i) measuring the cross-correlation of vocal ratio to instantaneous speed (Figures 1D, 2B, 3C, 4E), (ii) measuring call rates for different ranges of instantaneous speed (Figures 1E, 2F, 3B, 4G) and (iii) contrasting call rates (Figure 2G) or vocal ratio (Figure 4F) during episodes of staying in place (lingering) vs. moving between places (progressing).

- We obtained “vocal ratio” as a continuous representation of vocal production from each rat. For each time point, it measures the fraction of time that the rat spent emitting ultrasound in a short time window around it. It thus ranges from 0 (silence) to 1 (continuous vocalization within the time window). Examples of vocal ratio time series can be seen in the blue traces in Figures 1C, 2A, 4D. To calculate it, we first constructed a binary time series of 0.25 ms time step with value 1 at times when ultrasound emission was detected from the rat and 0 otherwise. We finally smoothed this vector by convolving it with a Gaussian window of full width at half maximum 0.25 s.
  For linear cross-correlation analysis, we interpolated both instantaneous speed and vocal ratio to a common time axis with 5 ms step. We then obtained the normalized cross-correlation between these two time series.
- For call rate vs. instantaneous speed, we divided the time in 25 ms bins, calculated mean instantaneous speed for each bin and grouped them in speed ranges. We then divided the total number of calls emitted within bins of each speed range by the total time spanned by those bins.
- In Figure 2G we calculated the mean call rate during episodes of lingering and progressing for rats in social or isolated sessions. In Figure 4F, we analyzed how the emission of USVs changed at the times that the rats started (left) or ended (right) their progressions. For this purpose, we obtained the times of these transitions for each rat and plotted the mean relative vocal ratio around them. Relative vocal ratio was obtained by dividing instantaneous by mean vocal ratio for each rat.

### Classification of calls

We classified the USVs emitted by rats in groups A and B during social sessions. We selected only calls with duration of over 10 ms and good signal-to-noise ratio (low mean entropy). One rat from group A had less than 200 USVs meeting these criteria and was not included in the analysis, leaving a total of 24001 calls for 7 rats (range 461–4576 calls per rat).

We developed a semi-automatic method to classify the USVs based on their spectrotemporal profile. The main features taken into account for each call were its mean fundamental frequency, its frequency bandwidth and whether its frequency had a tendency to rise or fall with time. Based on these properties, we clustered USVs in 4 classes. Class 1 was of stable intermediate frequency and low bandwidth such as “flat” calls. Class 2 was of maintained high frequency and bandwidth such as “trill” calls. The mean frequency and bandwidth of calls in Class 3 were intermediate to those of classes 1 and 2, and they had a tendency for their frequency to either rise or fall with time. Thus, this class included calls such as combinations of flat and trill segments (either “flat-trill” or “trill-flat”). Class 4 was of low mean frequency with a tendency to first fall and then rise, and was exclusively composed of the calls known as “step,” “split,” or “harmonic,” with their fundamental frequency momentarily jumping down to the ~30–35-kHz range with a visible second harmonic (call class names taken from Burgdorf et al., 2008; Ciucci et al., 2009; Wright et al., 2010).

In detail, we classified the calls by first representing each vocalization as a vector of its fundamental frequency vs. time and morphed all of them to a common length. We then performed singular value decomposition (SVD) of the calls from each rat and kept their projections in the first three directions, which approximately represented the main frequency of a call, its tendency to change frequency monotonically and its tendency to first rise and then fall or the opposite. For each call we also calculated bandwidth as the root mean square of the deviations from its mean frequency. We used these 4 values to cluster calls using the “mean shift” method (Comaniciu and Meer, 2002), from which the classes presented in Figure 3 emerged. Flat-trill combinations, because of their heterogeneity, usually came out divided into smaller clusters that we grouped together to form class 3.

### Statistical analysis

We had not planned contrasts between vocal production and locomotor activity when first designing the experiments. We have thus throughout the manuscript favored descriptive statistics and effect sizes over null hypothesis significance testing, as the interpretation of *p*-values is impaired for hypothesis that emerge from observation of the collected data (Simmons et al., 2011). We have nevertheless included significance testing as reference for all analyses. For assessing significance of speed vs. vocal ratio linear correlations, the variables were subsampled to 1 value every 2 s (small arena) or 5 s (large arena) to minimize spurious contributions from autocorrelation (Figures 1D, 2B, 3C, 4E).

## Results

### Vocalizations synchronize to locomotion at a subsecond time scale

We first performed synchronized ultrasound and video recordings from 8 adult male Long Evans rats interacting in pairs in a custom-built social arena (animal groups A and B, 4 rats each, 3 different pairings per rat, 15 min per session). In the arena, both rats could hear and smell each other in the dark across a 25 cm gap (Figure 1A). We reconstructed from the video recordings the position and instantaneous speed of locomotion time series for each rat. Rats were active throughout the sessions (mean ± S.D. distance traveled per rat per session: 40 ± 6 m; *N* = 8 rats), with their locomotion characterized by a fast alternation between periods of in-place exploration and short runs (see examples in Figure 1A and Supplementary Movie 1). Analysis of audio from a pair of overhead ultrasonic microphones allowed us to assign vocalizations to each rat with > 99% accuracy (see Figure 1B, Section Materials and Methods and Sirotin et al., 2014). Rats were highly vocal throughout the sessions, emitting only calls of the 50-kHz family (mean ± *S.D*. number of calls per rat per session: 1500 ± 800). Upon temporally aligning call times with instantaneous speed it became apparent that rats emitted many of their calls during locomotion (Figure 1B and Supplementary Movie 1). To quantify the relationship between these two behaviors we first treated both as continuous time series. We represented locomotor activity by the instantaneous speed of the rat and measured vocal production as “vocal ratio,” the fraction of time spent emitting ultrasound in a short time window, both signals smoothed with a Gaussian window of width 0.25 s. Upon overlaying, these two signals typically matched each other (Figure 1C). Cross-correlation analysis confirmed instantaneous speed and vocal production were positively correlated for all rats (Figure 1D; all rats *p* < 0.0001, linear correlation at zero lag). This correlation fell rapidly at lags of over a second, highlighting that both behaviors are intimately linked in time. The peak of the cross-correlation was consistently shifted from zero time, revealing that vocal production preceded the speed increase by about 140 ms (lag different from 0 with *p* < 0.0001, one sample *t*-test, *N* = 8 rats). As a result of this synchronization of vocal and locomotor behaviors, call rates increased as a function of instantaneous speed for all rats [Figure 1E; effect of speed *F*_(5, 35)_ = 59.5, *p* < 0.0001, repeated measures ANOVA].

### Social context modulates call emission

A rigid link between the emission of ultrasound and locomotion could suggest these sounds are not flexible social signals but are instead by-products of stride mechanics (Blumberg, 1992). If this was the case, any condition promoting locomotion should result in increased call rates and vice versa. Our experimental design included sessions where we recorded the same rats in the same arena but with no other rat present (“isolated” sessions). These sessions were interspersed with the ones recorded with pairs of rats (“social” sessions). We contrasted the results obtained during social vs. isolated sessions to dissect the effects of social context on vocal and locomotor behaviors. When recorded alone, rats could go through periods of active locomotion with little or no emission of USVs (Figure 2A, left). Rats could also be vocal in isolation, in which cases vocalizations showed synchrony with locomotor activity too (Figure 2A, right). In fact, the cross-correlation of instantaneous speed and vocal ratio obtained for isolated rats is equivalent to that shown for social sessions (Figure 2B, compare with Figure 1D; *p* < 0.0001 for 7 rats, *p* < 0.01 for 1 rat, linear correlation at zero lag). Crucially, all rats vocalized less during sessions in isolation (Figure 2C; *p* < 0.001, paired *t*-test, *N* = 8 rats). Total distance traveled was also reduced although to a lesser degree (Figure 2D; *p* < 0.01, paired *t*-test, *N* = 8 rats), so that the number of calls per distance traveled was higher in social recordings (Figure 2E; *p* < 0.001, paired *t*-test, *N* = 8 rats). Indeed, at equivalent instantaneous speeds, rats were vocalizing more in the presence of a conspecific than in isolation [Figure 2F; Effect of social setting *F*_(3, 28)_ = 3.50, *p* = 0.03; Effect of speed *F*_(5, 140)_ = 130.4, *p* < 0.0001; Interaction *F*_(15, 140)_ = 1.0, *p* = 0.4; Two-way repeated measures ANOVA]. Thus, social context differentially modulated locomotor and vocal behaviors, specifically promoting vocal production.

Rat spatial behavior is structured in distinct modes of locomotion. During exploration, rats alternate periods of moving fast between places (“progressing”) with others of staying in one location with only local movement (“lingering”; Golani et al., 1993). We extended our analysis by segmenting the locomotor behavior of the rats into progressing and lingering episodes (see Section Materials and Methods) and analyzing how the presence of conspecifics affected call rates for each. Call rates were higher in social settings for both spatial behaviors (Figure 2G; *p* < 0.001 for both progressing and lingering, paired *t*-tests, *N* = 8 rats). Thus, the presence of a conspecific promoted vocalizations during both fast locomotion and in-place exploration.

### Classes of ultrasonic vocalizations have different links with locomotion

Rat USVs of the 50-kHz family can be divided in classes based on their spectro-temporal profiles (as in Burgdorf et al., 2008; Wöhr et al., 2008; Wright et al., 2010). We developed a semi-automatic protocol to classify calls during social sessions (~24000 total calls, see Methods). We classified calls into four classes, as shown in Figure 3A. Class 1 included calls with little frequency modulation (such as “flats”). Class 2 consisted of calls of high frequency and frequency modulation (“trills”). Class 3 included those calls that combined the previous two elements (such as “flat-trills” and “trill-flats”). Finally, class 4 consisted of those known as “steps,” “splits” or “harmonic” (Burgdorf et al., 2008; Ciucci et al., 2009; Wright et al., 2010), with their fundamental frequency momentarily jumping down to the 30–35-kHz range with a visible second harmonic. Class 3 was the most prevalent while class 4 was consistently scarce (Figure 3A, inset). To assess whether the observed link between vocal production and locomotor activity was equivalent across call classes, we measured how call rate depended on the speed of the rat for each class. This relationship was different across call classes (Figure 3B). The rate of calls in class 3 (flat-trill combinations) steadily increased with the running speed of the emitter. The rate of calls from class 1 (flats) showed an increase that plateaued for high speed while calls in class 2 (trills) were not reliably correlated with speed across rats. Remarkably, the typically rare calls of class 4 (steps) showed a large rate increase specifically when the rats were moving at the highest speeds [Effects of speed: class 1, *F*_(5, 30)_ = 14.2, *p* < 0.0001; class 2, *F*_(5, 30)_ = 1.4, *p* = 0.28; class 3, *F*_(5, 30)_ = 21.6, *p* < 0.0001; class 4: *F*_(5, 20)_ = 22.7, *p* < 0.0001; all repeated measures ANOVA]. To further understand the precise synchronization of calls from each class with locomotor activity, we obtained the cross-correlations of instantaneous speed with the vocal ratio (as in Figure 1D) of each class (Figure 3C). Calls in classes 3 and 4 were synchronized to locomotor activity for all rats (with *p* < 0.0001 for each), those in class 1 in all but one rat (with *p* < 0.0001) and those in class 2 in only 3 of them (with *p* < 0.001).

In a separate set of recordings, we induced emission of 22-kHz USVs by presenting pairs of rats with domestic cat scent (Supplementary Figure 2). These alarm calls were not synchronized to locomotion events and their emission showed no positive correlation with instantaneous speed. This was in contrast with the robust positive correlation between 50-kHz USV emission and locomotor activity exhibited by the same rats.

### High vocal production is maintained throughout prolonged progressions

We did not specifically design the experiments described so far to study locomotion. Runs in the small arena were brief and of limited speed (top 95% quantiles: duration of progressions, 2.5 s; top speed of progressions, 0.36 m/s). Because of these limitations, it was hard to distinguish whether rats keep vocalizing throughout the whole duration of progressions or rather USVs mark only their onsets. We also wondered how running at higher speeds would affect vocal production. We thus set up a larger social arena where rats could display richer locomotor behavior. It consisted of two 2.7 m long parallel tracks separated by a 0.15 m gap (Figure 4A). We recorded in this arena four male and two female rats interacting in opposite-sex pairs with the female under hormonally induced estrus (group C, see Section Materials and Methods). We developed a new method to assign calls to the emitting rat based on the telemetric measurement of nasal respiration, which follows a characteristic pattern when rodents emit USVs (Sirotin et al., 2014). We detected USVs from three overhanging microphones distributed along the tracks (Figure 4B, top). We then automatically assigned each USV to the rat whose respiration was most compatible with vocalization at those times (Figure 4B, bottom, see Section Materials and Methods and Supplementary Figure 1). Rats in this large arena achieved faster and longer runs (top 95% quantiles: duration of progressions, 9.5 s; top speed of progressions, 0.74 m/s).

Both males and females were vocalizing at high rates during locomotion (Figure 4C and Supplementary Movie 2; compare with Figure 1B). During fast runs, rats could maintain remarkably high levels of vocal production, vocalizing on almost every respiratory cycle (see female in Figure 4B) and achieving vocal ratio levels of over 0.5 (Figure 4D, filled triangles). Again, rats could also vocalize while staying in place (Figure 4D, open triangles). Cross-correlation analysis confirmed that locomotor activity and vocalization were tightly synchronized for both males and females (Figure 4E, compare with Figure 1D; cross-correlations are wider because of the longer duration of runs in the large arena; all rats *p* < 0.0001, linear correlation). Now that rats were doing longer runs, we could evaluate whether they were maintaining high vocal production during their whole durations by aligning their vocal ratio to the onset and offset of each progression (Figure 4F). All rats abruptly increased their mean vocal ratio shortly before movement began and maintained it at high levels until shortly before it ended. We were also able to quantify in this arena that call rates are high over a large range of speeds, likely encompassing the transition from walking to trotting gait [Muir and Whishaw, 2000; Gillis and Biewener, 2001; Figure 4G, compare with Figure 1E; effect of speed *F*_(4, 20)_ < 0.0001, repeated measures ANOVA, *N* = 6 rats].

## Discussion

### The “locomotion by-product” hypothesis

By the end of the 1970's, Thiessen and collaborators studied ultrasonic signals emitted by the adult Mongolian gerbil. While they found their production to be modulated by social setting, dominance status, and olfactory cues from conspecifics (Thiessen et al., 1978), they were unable to demonstrate specific contributions of these ultrasounds to social behavior. They thus turned to a detailed description of motor behavior at the times of ultrasound emission for inspiration. What they found was a strong link between ultrasonic vocalizations and locomotion (Thiessen et al., 1980). At broad temporal scale, rates of ultrasounds correlated with locomotor activity. In detail, frame-by-frame video analysis revealed that gerbils typically emitted ultrasounds by the end of jumps, when their forepaws hit the ground. This left open the interpretation that ultrasounds were a by-product of forced exhalations after physical compression of the lungs. In 1992, Blumberg argued this was also the case for the brief ultrasounds emitted by many other small rodents, including the laboratory rat (Blumberg, 1992). He did so based on principles of locomotion and respiration biomechanics, links between locomotion and ultrasound emission scattered across the rodent literature and high-speed video observation of a pair of mating rats. He reported that many—but not all—ultrasounds were emitted as the female forepaws hit the ground after a hop, although he included no quantification of this. Overall, he favored the conclusion that the possible communicational roles of rodent USVs were severely constrained by their link to locomotion mechanics.

Several lines of research have since argued against the “locomotion by-product” hypothesis for the emission of rat USVs, by showing that modulation of their rates by behavioral and pharmacological treatments cannot be fully accounted for by accompanying changes in locomotor activity (Knutson et al., 1998; Burgdorf et al., 2000; Schwarting et al., 2007; Natusch and Schwarting, 2010). This subject has further fueled debate about the interpretation of animal vocalizations (Blumberg and Sokoloff, 2003; Panksepp, 2003) but has slowly vanished in the recent literature. Remarkably, despite the frequently observed positive correlation between USV rates and overall levels of locomotor activity, we are not aware of any study directly aimed at dissecting this relationship. In fact, when in 2011 we started our current program of quantitative analysis of rat behavior with high temporal resolution we were not intended on addressing this issue. The subsecond synchronization of USVs with locomotion bouts we are now reporting was also not evident from observing the rats, although it seemed clear that our rats were vocalizing more when they were active. Only after the data was processed and speed and vocal production time series were aligned (as in Figures 1B,C) did this relationship become clear.

So are USVs in the 50-kHz family mere by-products of body movements during locomotion? Several lines of evidence argue against this. (1) It is evident from our data that rats can both vocalize without movement and move without vocalizing. (2) Vocal production increases before locomotion begins. From the observation of videos with superimposed vocalizations, it seems that the rats start vocalizing as they are getting ready to start a progression (see Supplementary Movies 1, 2). A recent re-evaluation of gerbil behavior showed that their calls can also precede movement, occurring at the onset of jumps and thus not in synchrony with forepaw landing (Nishiyama et al., 2011). (3) We know that USVs require active adduction of the vocal folds by contraction of larynx musculature (Roberts, 1975a). Frequency modulated calls make a particularly strong case for a specific evolution of USV production mechanisms. These stereotyped modulations of ~10 ms period are produced by a matching rhythmic contraction of intrinsic laryngeal muscles (Riede, 2013), which points to the evolution of a dedicated pattern generator for trill calls within the larynx motor nuclei. Interestingly, we found that flat-trill combination calls were strongly correlated with locomotor activity, further supporting that the rats actively increase USV rates during movement. (4) Call rates at all speeds are modulated by social context. Previous works have reported that compounded USV rates recorded from pairs of rats more than doubled rates from animals recorded in isolation (Brudzynski and Pniak, 2002; Wright et al., 2010). However, these studies did not quantify locomotor activity, so that the increased number of calls could be following increased locomotor activity in social settings. In our experiments, the presence of another male resulted in only a moderate increase in mean speed but a robust increase in call rates. Crucially, rats were vocalizing more in social settings for equivalent speeds, so that even during fast locomotion vocal production was modulated by social context. (5) From observing the videos, it appears that our rats were mostly walking or trotting when moving, which is consistent with other reports locomotor behavior for the measured speeds (Muir and Whishaw, 2000; Gillis and Biewener, 2001). These gaits involve alternation of left and right limbs and would not exert as much pressure on the lungs on stepping as expected for galloping and hopping. We have recently reported a quantification of the detailed synchrony between respiration, USV emission and body movements during locomotion. We found that rats can emit 50-kHz USVs at all phases of the stride cycle, further arguing against a strict causal link between locomotion movements and vocal production (Alves et al., 2016).

### Is it really locomotion?

Rat behavior is dauntingly multidimensional, so there can always exist hidden variables driving an observed correlation. Vocalizations could be part of a composite behavior that includes locomotion. Conversely, vocalizations and locomotion could be directly linked, such that any social behavior that includes locomotion will evoke high rates of USVs. A striking feature of the observed correlation is its temporal precision. Vocal production rises and falls within a few hundreds of milliseconds of the onset and offset of movement. Therefore, our data does not merely reflect the known correlation between call rates and broad levels of arousal or activity, but a faster link of vocal and motor behavior. If vocalizations are linked to locomotion through intermediate variables, all of these must be precisely synchronized.

### Locomotion and call properties

Call rates in our small arena were unusually high for adults, with rats emitting on average 1.5 calls per second over 15 min in social sessions (compare with ~0.15 Hz for 10 min in Brudzynski and Pniak, 2002 and ~0.35 Hz for 20 min in Wright et al., 2010). The most abundant call type was the combination of flat and trill segments. This was also unusual as other reported recordings of pairs of interacting male rats are dominated by trill calls, both for juveniles and adults (Wright et al., 2010; Himmler et al., 2014). In social primates, call rates increase and acoustic properties vary with distance between individuals (Boinski, 1991; Rendall et al., 2000). Call properties in our recordings could be a result of keeping the rats interacting without physical contact. Intriguingly, calls of different classes showed varying relationships with locomotion speed. Flat-trill combinations were consistently synchronized with locomotion and their rates steadily increased with running speed. On the contrary, locomotion variably modulated the emission of pure trill calls. The typically rare “step” calls were remarkable in that their rates increased by an order of magnitude during high-speed runs. If rat USVs are indeed produced by an aerodynamic whistle mechanism, their fundamental frequency at each time will depend on mechanical factors like the exact geometry of the larynx and airflow through it (Brown, 1937; Roberts, 1975b; Howe, 1998). Indeed, different spectral profiles are distinguished by larynx muscle activity and subglottal pressure (Riede, 2014). Geometry and airflow could, in principle, also be dependent on body/neck posture and pressure on the lungs, which are affected by locomotion. We cannot thus rule out the possibility that the appearance of “step” calls at high speeds reflects mechanical strains facilitating a frequency jump to a rarer acoustic mode with resonance at ~30-kHz (Howe, 1998). On the other hand, different combinations of flat and trill elements require distinct coordinations of larynx muscles, so the opposed correlations of trill and flat-trill with speed likely reflects alternative vocal motor programs being favored during locomotion. More research is needed to understand how universal these specific links of call classes with locomotion are across sexes, ages, and behavioral/emotional state.

In clear contrast with the results obtained for 50-kHz USVs, we observed that emission of 22-kHz alarm calls was not positively correlated with locomotor activity, but was instead associated with low or no locomotor activity. This is consistent with previous reports associating emission of these calls with freezing behavior (Kim et al., 2010; Parsana et al., 2012). Interestingly, we noted that emission of shorter bouts of these calls could actually happen as the rat was moving in the arena.

### Neural systems linking vocalizations with locomotion

It follows from our results that motor nuclei for locomotion and vocalization in the rat are frequently recruited within a few hundreds of milliseconds of each other. Which could be the upstream brain systems behind this coordination? Changes in neuronal activity across the brain can precede the initiation of voluntary movements by hundreds of milliseconds (Vanderwolf, 1969; Fuhrmann et al., 2015; Roseberry et al., 2016). Activations of the mesolimbic dopaminergic pathway trigger behaviorally activated states that include increases in locomotor activity and in 50-kHz USV rates (Fu and Brudzynski, 1994; Burgdorf et al., 2000, 2001; Thompson et al., 2006). However, locomotor activity in those cases does not fully account for the measured increases in USV rates (Burgdorf et al., 2000, 2001). Furthermore, we observed that a social context resulted in a generalized increase in vocal production, both during progressing and lingering episodes. Mesolimbic activity could be linking arousal and USV rates at the seconds time scale while modulating the expression of a subsecond synchronization between locomotor and vocal motor activity in specific brain nuclei. Activity of neurons along the direct pathway of the basal ganglia to the mesencephalic locomotor region triggers locomotion in mice with latencies in the hundreds of milliseconds (Ryczko and Dubuc, 2013; Roseberry et al., 2016). It would be interesting to test whether activation of this circuit evokes concomitant USVs in rats and how this depends on social and emotional context.

### Contributions of locomotion-linked vocalizations to rat social behavior

Rat 50-kHz USVs have been proposed to function as “contact calls,” as they can both coincide with acute separation from conspecifics and trigger approach behavior (Seffer et al., 2014). Contact calls in primates and birds play a crucial role in maintaining cohesion and synchronizing movements of the social group (Boinski, 1993; Rendall et al., 2000; Marler, 2004; Radford, 2004; Kondo and Watanabe, 2009). In our recordings, rats that were actively vocal would always vocalize while moving from one place to the other. In principle, rats could then keep track of the position and speed of neighboring partners by listening to their vocalizations alone. Unlike the 22-kHz alarm calls, USVs of the 50-kHz family are brief and with rapid changes in frequency, making them theoretically easier to locate (Marler, 1955). Indeed, rats can track the origin of 50-kHz USVs, as evidenced by the fact that they approach their sources in playback experiments (Wöhr and Schwarting, 2007; Seffer et al., 2014; Willadsen et al., 2014). One contribution of this family of USVs could thus be the social coordination of spatial behavior. The facts that (a) rats vocalize while running and (b) rats approach the sources of USVs, suggest that these calls could support following/chasing behavior in the right social settings. Males are known to chase females during courtship behavior in large-enough environments (Adler and McClintock, 1978) and pups have been reported to closely follow their mothers (Barnett, 1975). In mice, both males and females increase the rates of their ultrasonic calls during chases (Neunuebel et al., 2015). While odors most likely contribute to approach behaviors, vocalizations could prove important for allowing fast spatial interactions in these nocturnal animals. It has not escaped our notice that high rates of ultrasonic vocalizations during locomotion could aid the rats navigate in the dark through echolocation. However, the few studies supporting that rats can indeed echolocate did not find ultrasonic vocalizations to be involved (Rosenzweig et al., 1955; Riley and Rosenzweig, 1957; Chase, 1980). In addition, we found that rats running at the same speeds vocalize more in the presence of conspecifics, which points to the involvement of these signals in social behavior.

## Conclusions

The laboratory rat is an invaluable model for mammalian behavior as it displays flexible, complex behaviors and is ideally suited for systems neuroscience approaches, biochemical analysis and pharmacological interventions (Whishaw and Kolb, 2004). Because of this, the realization that rats display rich vocal production in the ultrasonic range (Anderson, 1954; Sewell, 1967) raised the promise of establishing the species as a useful tool for the dissection of animal communication systems and behavior and, potentially, fundamental aspects of human vocalization. Fulfilling this promise requires that we uncover what functions these calls play in behaviors that rats can express in controlled laboratory settings.

To advance our understanding on the roles of ultrasonic calls in the social behavior of the laboratory rat, we performed synchronized audio and video recordings of pairs of animals interacting at a short distance, together with quantitative analysis at high temporal resolution. Both male and female adult rats consistently synchronized their locomotion with high rates of 50-kHz USV emission with subsecond precision. Call rates were, thus, positively correlated with instantaneous speed. Remarkably, different call classes within this family of USV where differentially modulated by locomotor activity.

Links between USV rates and locomotor activity have been suspected before (Sales, 1972; Thomas and Barfield, 1985; Blumberg, 1992). Despite this, both variables were never, to our knowledge, quantified together with high temporal resolution and their association was all but dismissed based on observations that changes in USV rates cannot be fully explained by changes in locomotor activity levels (Knutson et al., 1998; Burgdorf et al., 2000; Schwarting et al., 2007; Natusch and Schwarting, 2010). Our results bridge this controversy by showing that 50-kHz USV have indeed intimate links with locomotion, but are not mechanical by-products of it since rats can vocalize without locomotion and the rates at which they vocalize at any speeds can be modulated by other conditions such as social context. The realization that a large fraction of the variance in rat 50-kHz USV call emission can be linked to locomotor activity suggests it could be beneficial to include video tracking in future experiments in the field. Even if movement were not of interest, including it in the analysis would allow the experimenter to obtain cleaner correlations of USVs with other variables at both broad and detailed temporal scales. As we previously found for alarm calls (Assini et al., 2013), emission of calls within the 50-kHz family can be linked both to emotional state at broad temporal scales and to specific behaviors with sub-second precision. Experimental interference on ultrasonic vocalization emission or perception has not resulted in robust disruption of rat social behaviors such as mating (Thomas et al., 1981; White and Barfield, 1987; Snoeren and Ågmo, 2013, 2014a,b; Ågmo and Snoeren, 2015) or dominance (Takahashi et al., 1983; Thomas et al., 1983). Of note, most studies were conducted with animals interacting in small environments, where any spatial information carried by vocalizations would be redundant. Experiments where space is a factor will be needed to test the hypothesis that some of the behavioral roles played by rat ultrasonic vocalizations are expressed in the social coordination of spatial behavior.

## Author contributions

DL and ME designed the experiments, set up the recordings, collected data, programmed the analysis routines and analyzed the data. DL wrote the manuscript and prepared the figures.

### Conflict of interest statement

The authors declare that the research was conducted in the absence of any commercial or financial relationships that could be construed as a potential conflict of interest. The reviewer AJR and handling Editor declared their shared affiliation, and the handling Editor states that the process nevertheless met the standards of a fair and objective review.
