# Supplementary material for: Rats Synchronize Locomotion with Ultrasonic Vocalizations at the Subsecond Time Scale
Source: Front Behav Neurosci. 2016 Sep 29;10:184. doi: 10.3389/fnbeh.2016.00184 (PMC5040720; doi:10.3389/fnbeh.2016.00184)
Supplement: Supplementary file 3 [file Presentation1.pdf]

Supplementary Material

Rats Synchronize Locomotion with Ultrasonic Vocalizations at the Subsecond Time Scale

Laplagne, Diego A.[1]*, Elias Costa, Martín

*** Correspondence:** Diego A. Laplagne, dlaplagne@neuro.ufrn.br

1 Supplementary Movies

Supplementary Movie 1. Movie (.mp4) of a male rat from group B during part of a session in the small arena. Position of the rat obtained from video tracking is marked by the blue circle. At every frame when the rat starts emitting a vocalization, its frequency profile is drawn at the rat's position and maintained for 250 ms of recording. USVs are colored according to call class (1: cyan, 2: yellow, 3: green, 4: white, unclassified: black). Frame number and recording time can be read on the top-left of the image. Bottom panel shows instantaneous speed (black, m/s) and vocal ratio (red) for a period spanning 2.5 seconds before and after the current frame (time 0).

Supplementary Movie 2. Movie (.mp4) of a female rat during part of a session in the large arena. Details same as movie 1 except USVs were not classified and are shown in red.

2 Supplementary Figures

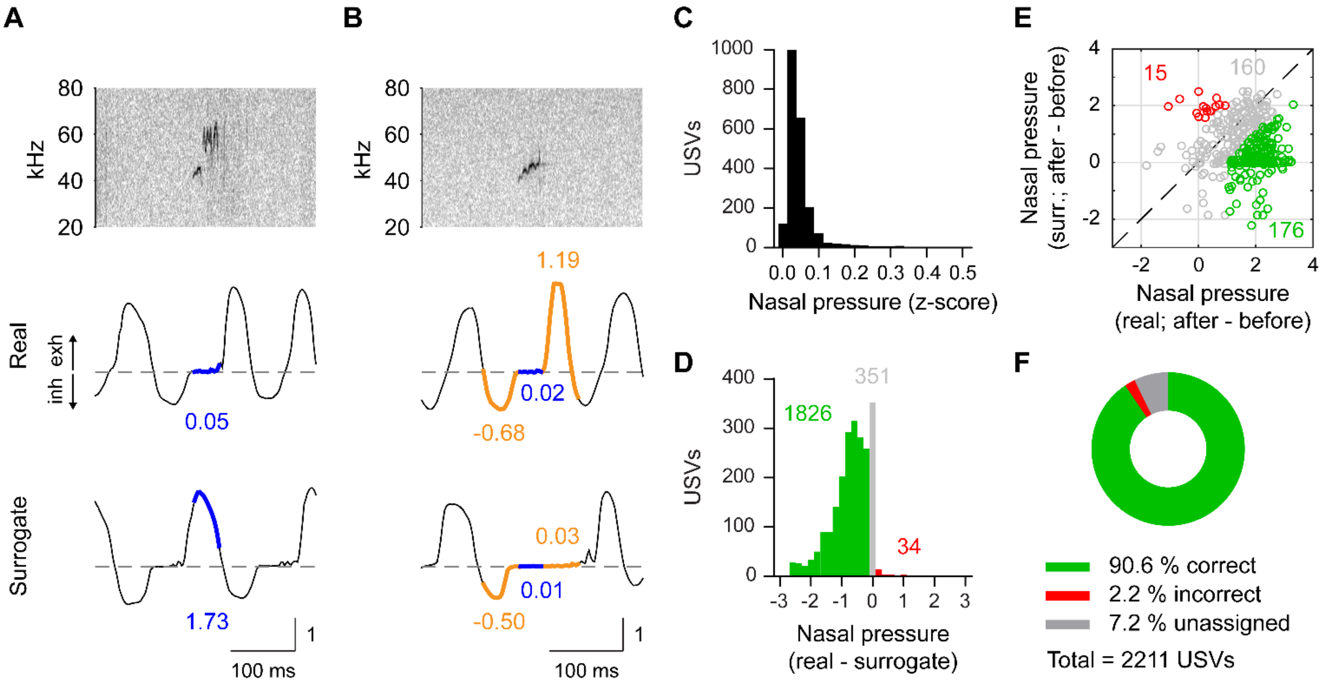

Supplementary Figure 1. Assignment of USVs in the large arena based on respiration.
In recordings of pairs of rats in the large arena, we automatically assigned each USV detected from the overhanging microphones to one of the rats by comparing their respiration at the time of the call, as explained in Methods and seen in Figure 4B. To estimate the accuracy of this protocol, we recorded a rat behaving alone in one side of the arena, such that we know that all the calls detected were emitted by it (22 minutes, total 2211 USVs). Errors in the assignment of a USV would arise in cases where, by chance, the respiration from a non-vocalizing rat at the time of the vocalization fits the assignment criteria better than that of the emitting rat. To simulate the presence of another rat, we created a surrogate respiration signal by circularly shifting the real respiration signal of the rat by a random amount of time and ran the assignment protocol comparing the real and surrogate signals (ideally, 100% of the USVs should be assigned to the real rat). Panels **A** and **B** show two examples of USVs (sonogram on top), original respiration of the rat (middle) and surrogate respiration (bottom) at that time. Respiration traces are nasal pressure (z-scored). As it can be seen in the real data, USVs occur during near-zero (atmospheric) nasal pressure. This is quantified in panel **C**, a histogram of mean absolute nasal pressure during the emission of each USV. The method first attempts to assign each USV by comparing the mean absolute pressure during its emission from both rats (blue traces in A and B): if one is lower than the other by over 0.1, the call is assigned to the rat with the lowest value. In A, 0.05 in the real respiration is much lower than 1.73 in the surrogate and, thus, the call is correctly assigned to the real rat. As shown in panel **D**, 1826 USVs were correctly assigned in this way to the real rat (green) while 34 were wrongfully assigned to the surrogate (red). The 351 cases with pressure differences under 0.1 (gray) remain unassigned in this step. Panel B shows an example of this, where the pressure was low in both respiration signals. Typically, USVs are immediately preceded by an inhalation and followed by an exhalation (see the real respiration here and analysis in Sirotin et al., 2014). In a second effort to resolve these unassigned calls, the algorithm subtracts the mean pressure before the USV from that after it in the respiration from each rat (50 ms windows, orange traces in B). If for one rat this value is both larger than 1.0 and larger than that of the other rat by at least 1.0, the call is assigned to it. The USV in panel B is correctly assigned to the real rat because 1.19 - (-0.68) > 1.0 and 1.87 is larger than 0.53 by more than 1.0. As shown in panel **E**, of the 351 previously unassigned calls, 176 were now correctly assigned to the real rat, 15 wrongfully assigned to the surrogate and 160 remained unassigned. Overall, 92.8% of the detected USVs were assigned with an accuracy of 98.2% (panel **F**). Repeating the analysis with different random time shifts for the surrogate respiration resulted in very similar accuracies.

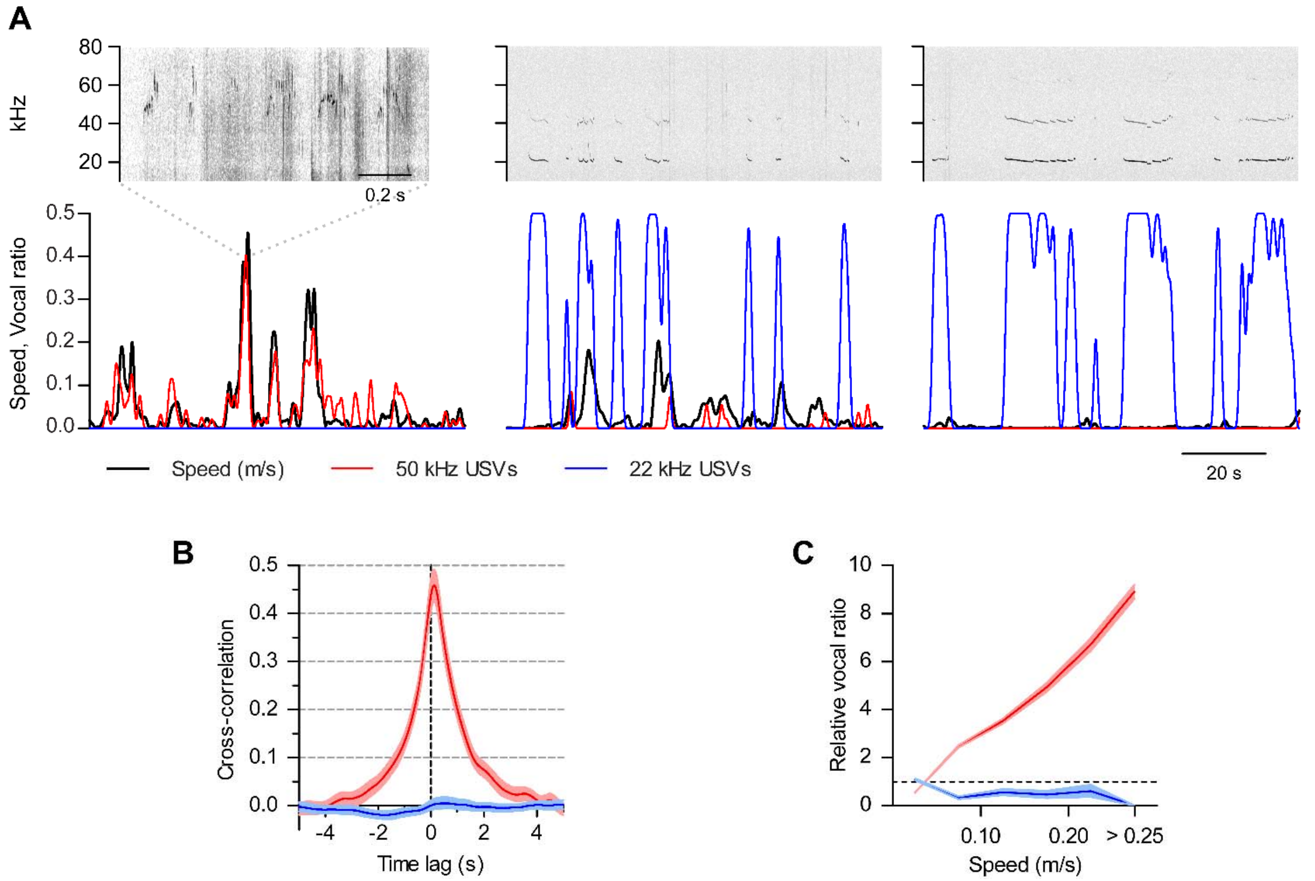

Supplementary Figure 2. Lack of synchronization of '22 kHz' vocalizations with locomotion.
We induced the emission of 22-kHz USVs, also known as alarm calls, by introducing the scent from soiled domestic cat litter into the recording room. This previously unpublished data is from two adult male rats included in Sirotin et al., 2014, recorded in two different sessions in the arena here called 'small arena' and in the presence of another male on the other side. We labeled all recorded USVs with mean fundamental frequency under 28 kHz as putative 22-kHz calls and verified each one by visual inspection (integrated duration of 22-kHz USV emissions for each rat were 46 and 42 s). All other USVs were labelled as 50-kHz.
A. Three periods (45 s each) from the same recording session of one of the rats: before introducing the cat scent (left) and 2.6 and 3.7 min after introducing it (middle and right). Graphs at the bottom show instantaneous speed of locomotion (black) and emission of 50-kHz USVs (red) and 22-kHz USVs (black) (emission of USVs measured by vocal ratio, as in Figure 1C; vocal ratio for 22-kHz was brought to the 0-0.5 range for clarity). Sonograms on top show the emitted vocalizations. The one on the left is an expansion of a ~ 1 s period indicated by the dotted lines (these 50-kHz calls are embedded in bedding noise as the rat was running at the time). Sonograms in middle and right are of the whole periods and are aligned to the traces on bottom. Before exposure to cat odor, the rat is emitting bouts of 50-kHz USVs aligned to locomotor activity. Shortly after exposure, it begins emitting 22-kHz calls in short bouts, interleaved with some movements and emission of 50-kHz calls. Note that the rat can move while emitting 22-kHz USVs. Later, the rat stays almost immobile as it exclusively emits long bouts of 22-kHz USVs.
B. Cross-correlation of instantaneous speed and emission of 50-kHz (red) or 22-kHz (blue) USVs (vocal ratio; mean and 95% CI). Emission of 50-kHz—but not of 22-kHz—USVs synchronizes with locomotor activity (compare with Figure 1D). For this analysis, we combined the recordings from both rats (90 minutes total) and run cross-correlations on each 30 s time window. We then estimated the mean and 95% CI from 1000 re-samples of these. (Efron and Tibshirani, 1986).
C. Mean Emission of 50-kHz (red) or 22-kHz (blue) USVs vs. instantaneous speed of locomotion, combining the data from the two rats (vocal ratio; mean and 95% CI). Emission of 50-kHz—but not of 22-kHz—USVs shows strong positive correlation with speed. Because of the long duration of the 22-kHz USVs, call rate (as used in Figure 3B) is not a good instantaneous measure of emission. We thus measured the mean vocal ratio at each 25 ms time bin instead. We estimated the mean and 95% CI from 1000 re-samples of the vocal ratio values for each speed bin.

3 References

Efron, B., and Tibshirani, R. (1986). Bootstrap methods for standard error, confidence intervals, and other measures of statistical accuracy. *Stat. Sci.* 1, 54–75. doi:10.1214/ss/1177013817.
